# Development of a Multifunctional Needle for Percutaneous Heart Biopsy and Cell Therapy. A Technical Note

**DOI:** 10.5935/1678-9741.20160092

**Published:** 2016

**Authors:** Nathan Valle Soubihe, Agnes Afrodite S. Albuquerque, Livia Arcêncio, José Antonio Thomazini, Andre Schmidt, Paulo Roberto B. Evora

**Affiliations:** 1 Department of Surgery and Anatomy, Faculdade de Medicina de Ribeirão Preto da Universidade de São Paulo (FMRP-USP), Ribeirão Preto, SP, Brazil.; 2 Department of Internal Medicine, Faculdade de Medicina de Ribeirao Preto da Universidade de São Paulo (FMRP-USP), Ribeirão Preto, SP, Brazil.

**Keywords:** Stem Cells, Biopsy, Myocardium

## Abstract

Validation of transendocardial injection as a method for delivering therapeutic agents to the diseased heart is increasing. Puncture heart biopsies should re-emerge as a possible alternative method to allow access to the myocardium and implantable biomaterial for cell therapy. Therefore, this work aims to present a percutaneous puncture device for biopsy and intramyocardial biomaterial injection, standardize the technique and attest to the safety of the method. The adaptation consists of creating myocardial microlesions that allow for better fixation of stem cells. The objective of this technical note covers only the development of the needle and the histological quality of the biopsies. It has not been used in humans yet.

## INTRODUCTION

The search for safe and effective methods of obtaining intact infarction fragments has inspired countless authors and has been the subject of several studies. Over the years, the development of different procedures that allow the obtaining of cardiac tissue fragments went through several stages, evolving from open myocardiectomy and procedures with puncture needles to endovascular catheters. Despite being in disuse today, procedures with puncture needles are particularly important for offering access to the myocardium and the left ventricular cavity. Thus, puncture heart biopsies should re-emerge as a possible alternative method to allow access to the myocardium and implantable biomaterial for cell therapy. Therefore, this work aims to present a puncture device for biopsy and intramyocardial biomaterial injection, standardize the technique and attest to the safety of the method. The adaptation consists of creating myocardial microlesions that allow for better fixation of stem cells.

The objective of this study covers only the development of the needle and the histological quality of the biopsies. A previous note was published in this journal^[^^1^^]^.

## DESCRIPTION OF TECHNOLOGY

**Fig. 1:**
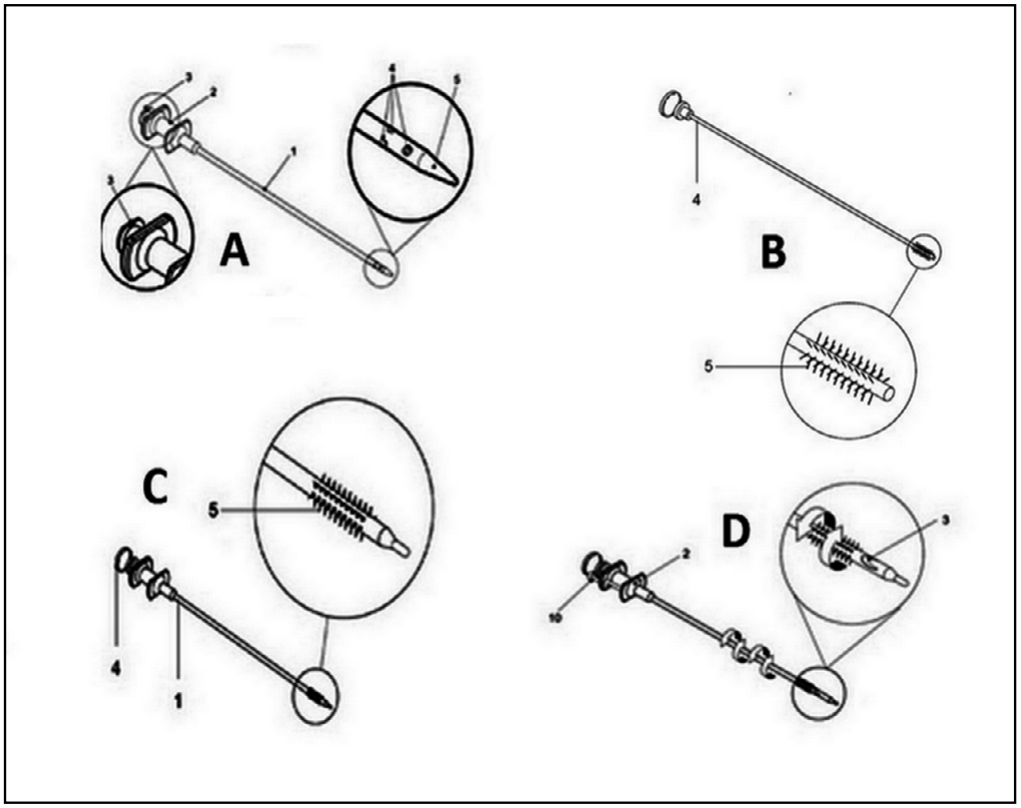
Instrument Design. **A -** External needle; **B -** Lateral holes for infusion; **C -** Blunt tip punch + brush mandrel; **D -** Internal mandrel.

**Fig. 2:**
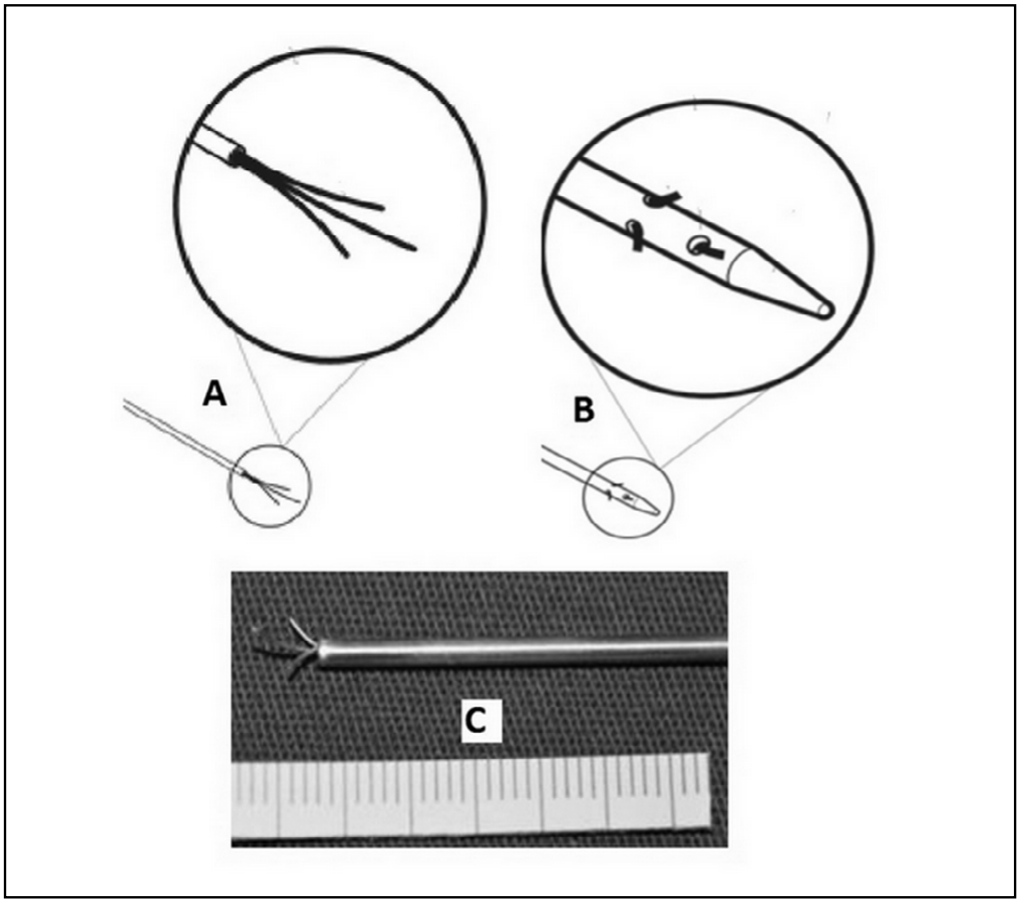
Instrument Design. Flexible metallic bristles (“Feather mandrel” as an option to the brush mandrel).

The instrument for puncturing and injecting biological material is composed of an external needle (1), called coupling infusion, which contains a blunt tip (2) and multiple 0.5 mm diameter holes (3) at its end. Internally, it is fitted with a blunt mandrel that can be mobilized to fill the lateral holes, occluding or releasing them. The procedure for producing microlesions is done by replacing the blunt mandrel with a brush-mandrel (4), structurally designed to fill the holes by exteriorizing the small bristles (5) (Figure 1). The instrument is equipped with a locking mechanism that allows its complete mobilization as one single unit for microlesions. It can also be used simply as an external needle, thus becoming an instrument for biological injection. It was designed with an optional feather-mandrel (Figure 2).

**Fig. 3:**
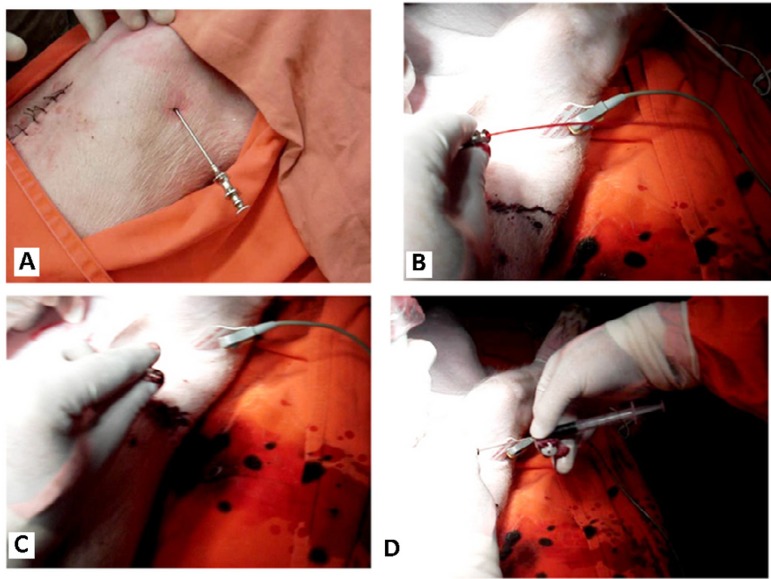
Percutaneous puncture technique. **A -** The percutaneous needle is introduced at a point 1 to 2 cm away from the apex of the heart; **B -** it reaches the left ventricular cavity (pulsed blood jetting); **C -** A slight and slow retreat of the needle until the blood jetting stops means that the muscular wall of the left ventricular myocardium completely occluded the holes; and **D -** The operator introduces the mandrel with bristles for scraping so as to create microlesions. After withdrawing the brush mandrel, the syringe containing the biological material to be infused into the previously scarified myocardium can be engaged.

**Fig. 4:**
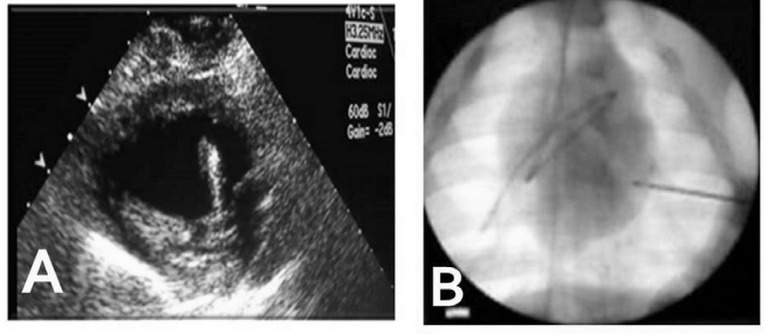
The instrument is easily visualized. **A –** Echocardiography; **B –** Radioscopy.

The percutaneous needle is introduced at a point 1 to 2 cm away from the apex of the heart (Figure 3A) and it reaches the left ventricular cavity (Figure 3B). A slight and slow retreat of the needle until the blood jetting stops means that the muscular wall of the left ventricular myocardium completely occluded the holes (Figure 3C). It is noteworthy that the distribution of these holes on the side of the needle never exceeds the minimum width of the common ventricular wall, allowing a slow and accurate withdrawal movement of the needle. Blocking the blood flow through the proximal opening without the mandrel is an accurate indication of the perfect location of the side holes in the ventricular wall. Then, the operator can introduce the mandrel with bristles for scraping so as to cause microlesions (Figure 3D). After withdrawing the brush mandrel, the syringe containing biological material to be infused into the previously scarified myocardium can be engaged. The instrument is easily visualized by either echocardiography (Figure 4A) or radioscopy (Figure 4B).

**Fig. 5:**
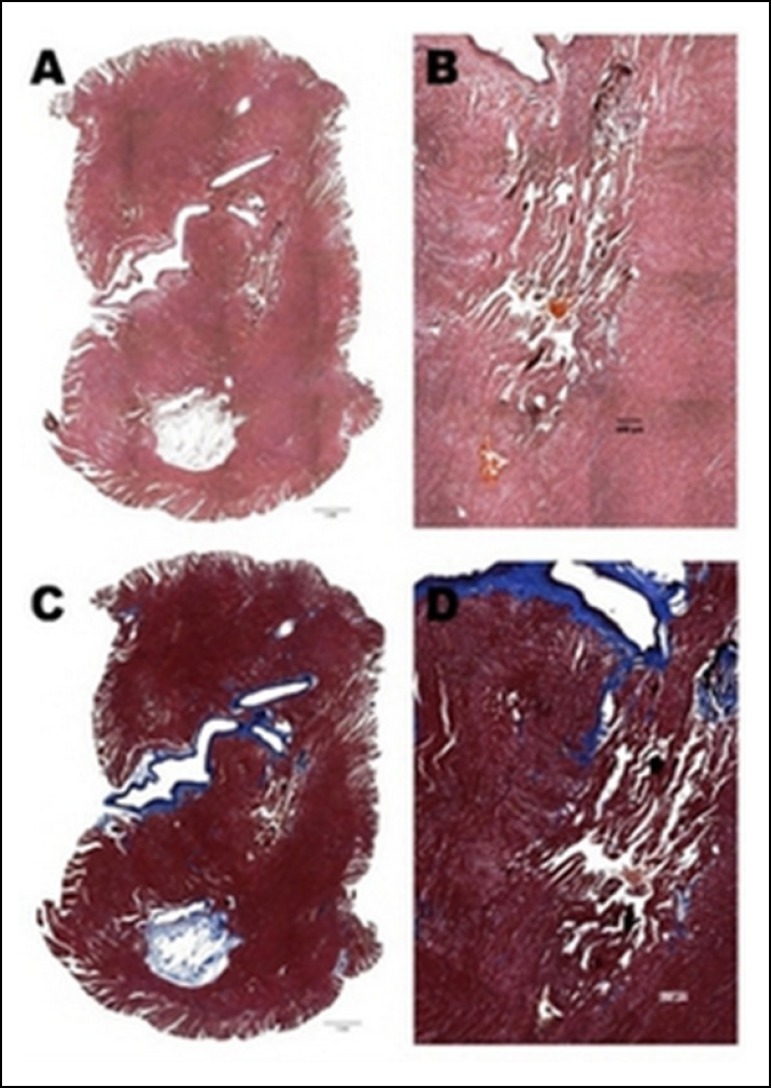
Histological samples. The results are presented through microscopic aspects of the heart. **A** and **B -** Hematoxylin-eosin; **C -** Masson; and **D -** Evans blue.

The technique has been tested in *in vivo* pig models and it has shown itself to be feasible and safe. The results are presented through microscopic aspects of the heart (Hematoxylin-eosin, Masson, and Evans blue) (Figure 5).

## DISCUSSION

The initial motivation for this work was the proposed experimental procedure for injection of stem cells in the myocardium of animals and humans through a multifunctional transthoracic puncture needle. The design followed two classical principles: 1) safety, in order to avoid accidents; and 2) technical capability of reaching the wall of the heart muscle, ensuring the biomaterial was left there, and preventing its release into the ventricular cavity or between the epicardial and pericardial. This procedure tends to become an intervention with increased accuracy and increased safety due to multiple punctures and a greater extent of myocardial tissue covered with cell therapy. In addition, direct injection as adjunctive therapy to revascularization surgery is a viable proposition, but it caters to a select group of patients referred to surgical revascularization immediately after an acute ischemic event. Intramyocardial injection during thoracotomy, in the surgical treatment of coronary artery disease, is a real possibility^[^^2^^-^^5^^]^.

## CONCLUSION

In conclusion, the new instrument is designed to be a multifunctional central feature: 1) It allows the operator to access the left ventricular cavity through the transthoracic without risk of injury (perforation) of the coronary arteries; 2) It allows for myocardial laceration of the muscle fibers by severing them and ripping the myocardium, thereby generating muscle microlesions through its arbor with bristles and promoting an "inflammation beneficial to the cell transplant process"; 3) The need for multiple punctures in the heart muscle to infuse standard biological material while performing cell therapy made us aware that the percutaneous needle could generate greater technical difficulty, so we propose that it be used with the surgical technique of video thoracotomy; and 4) The device should be used for intraoperative stem cell injections, but it has not been used in humans yet.

**Table:** 

**Authors' roles & responsibilities**	
NVSJ	Original idea and design of the project; final manuscript approval
AASA	Technical support; final manuscript approval
LA	Writing and formatting of the text; final manuscript approval
JAT	Histological study; final manuscript approval
AS	Project planning; final manuscript approval
PRBE	Study design and writing of the paper; final manuscript approval
